# A Case of Extra-Uterine Pregnancy of Four Months, Delivered per Uterus

**Published:** 1891-02

**Authors:** J. W. Carhart

**Affiliations:** Lampasas, Texas


DANIEL'S

### TEXAS MEDICAL JOURNAL.

A Representative Organ of the Medical Profession, and an Exponent of Rational Medicine; devoted to the Organization, Advancement and Elevation of the Profession in Texas.

PUBLISHED MONTHLY.—SUBSCRIPTION $2.00 A YEAR.

Vol. VI. AUSTIN, FEBRUARY, 1891. No. 8.

### Original Articles.

CONTRIBUTED EXCLUSIVELY TO THIS JOURNAL.

*The Articles in this Department are accepted and published with the understanding that we are not responsible for, nor do we indorse the views and opinions of the writers by so doing.*

For Daniel's Texas Medical Journal.

### A CASE OF EXTRA-UTERINE PREGNANCY

### Of Four Month's Gestation, With Delivery Per Uterus; —Recovery.

BY J. W. CARHART, M. D., LAMPASAS, TEXAS.

[Read at Austin District Medical Society, December 9, 1890, and ordered published in the JOURNAL.]

I WAS called to see Mrs. V., wife of Rev. H. V., on August 19, 1890, and on digital examination per vaginam, I found a tumor about as large as a hen's egg, or larger, on the right side of the uterus, and in the region of the right fallopian tube, but as it seemed to me, a little lower than the tube, involving, as I thought, the broad ligament.

I could not detect fluctuation at the time, or inflammation, and there was no history of inflammation, but the lady complained of constant pain, worse when she was on her feet, extending, at times, down the right thigh. A distinct sulcus was clearly definable between the tumor and the fundus uteri. A portion of the tumor seemed to crowd somewhat, the bladder on the right side, and she was troubled with frequent desire to micturate. I was somewhat at a loss as to the real nature of the case in view of the absence of inflammatory conditions, but ventured to exclude tubal pregnancy for the time, and treated the case as one of lymphaginitis,

I accordingly used the battery with the negative pole in closest proximity to the tumor, using a light and comfortable current. I also used the glycero-cotton tampon in tbe vagina, and painted externally with tincture of iodine. This treatment was continued until September 22, nearly every day. I made careful digital examination at comparatively long intervals, so that I would be more able to detect any change in the size of the tumor.

On September 28 I made careful examination of the tumor, its size, position and shape, and was convinced that I had a case of tubal pregnancy to deal with. The lady was thin in flesh, relaxed, and somewhat anæmic, and the outlines of the tumor could readily be made out and I could get ballotment.

The tumor was much larger than on the occasion of previous examinations; it was irregular, or nodular in shape, and I fancied that I could distinguish the head of the fœtus. The depression between the fundus uteri was still distinct. The fundus of the womb was slightly enlarged, but the neck was long and slim, and I could readily pass a Peaseley's sound into the womb, to the fundus, without the least obstruction.

Her last menstrual period was on June 19, 1890. I told the lady that I had treated her case as long as I was willing to, without counsel, and requested her to have her husband call at my office. He called towards night of the 28th of September, when I informed him of my conclusion and of the gravity of the situation, and requested counsel. I suggested Drs. C. M. Ramsdell and J. W. Hamilton, who were entirely acceptable to the parties, and on the morning of the 29th we met at the patient's residence, when both my consultants made careful and thorough examination, without anæsthetic, which was not necessary, and the both confirmed my statements with regard to the tumor, but were not agreed as to tubal pregnancy. They thought that if an aspirating needle were introduced possibly pus might be evacuated; at the same time admitting the difficulty in the case in view of the absence of inflammatory conditions and of deciduous discharges common in tubal pregnancy. They thought inflammation might have existed, prior to my first examination, or subsequently, without being recognized. Accordingly, with thorough antiseptic precautions I introduced an aspirating needle into the tumor, through the vault of the vagina. The needle was connected by means of rubber tubing to a vacuum chamber. When the needle had penetrated to the depth of about three-quarters of an inch, I distinctly felt resistance, and so stated to my consultants. Some blood was withdrawn but no amniotic fluid. Dr. Hamilton thought I had struck a multilocular cyst, the needle passing through one sack and striking the walls of another.

From this time on I had the case under observation but did not treat the lady until October 15, when I visited her for the purpose of using the aspirator again, to see if I could not get amniotic fluid. On examination I was so fully satisfied that it was tubal pregnancy that I decided not to aspirate. On October 19, I was summoned hurriedly to her bedside, and informed that pains came on at about two o'clock that evening and lasted two or three hours, when there was a gush of blood, and severe flooding followed for awhile, but ceased just before my arrival, at about 5 o'clock.

As there seemed nothing for me to do especially, I left, with instructions for the patient to keep perfectly quiet in bed and to notify me should there be any new developments whatever.

The following day, October 20, I took Dr. Hamilton with me, and in view of the new developments he concurred with me in the diagnosis of tubal pregnancy. The depression between the tube and uterus, though yet distinct, had lessened, and the fundus uteri had enlarged somewhat, probably from the presence of blood clots.

Assuming that there was a possibility of the fœtus passing *per*

*vias naturales*, we introduced into the womb a Barnes' bag, with a silk cover to control dilatation, and by the use of an Allen's surgical pump, dilated to the full capacity of the bag. All the tissues of the patient seemed lax, and there was not sufficient complaint of pain to justify an anæsthetic.

We allowed our patient to rest until night when we visited her again. Dr. Hamilton advised a more thorough dilatation at that time, and an effort to reach the fœtus and attempt to deliver. I advised waiting until the following morning, resolved then to do abdominal section, if there were no contra indications.

At an early hour in the morning of the 21st Mr. V. came in haste and excitement and said that there was something protruding from the "birth-place" three or four inches long. I was soon at the bedside and found it to be a loop of the prolapsed cord. I dispatched Mr. V. for Dr. Hamilton. The tumor had manifestly lessened, and had become one with the fundus. The neck of the womb was shortened,—the canal patulous, and by a little effort I was able to detect the feet and succeeded in delivering the fœtus before Dr. Hamilton's arrival.

In a short time after, he delivered the after-birth, which was evidently in the tube, which could be felt to contract, as the placenta passed out into the womb. The placenta showed extensive fatty degeneration. The fœtus was well formed and was five and a half inches in length, and of four month's gestation. The lady has made a slow but uneventful recovery.

She is tall but slight of build,—light compleixon and about twenty-five years of age, and has given birth to two children at full term—no miscarriages except the one here detailed. Her former pregnancies were entirely normal--no unusual distension on the right side, thus excluding the possibility of unicorn uterus.

There is now, and has been, for months, sugar in her urine, for which I am giving her Cleman's solution of bromide of arsenic, and have her on anti-diabetic diet.

I am not at this time disposed to go into the literature of this subject—which, by the way, is limited—farther than to call your attention to a paper published in one of our Texas medical journals, by the late Dr. Lowry, of San Antonio (see February No.

DANIEL'S TEXAS MEDICAL JOURNAL, 1890), in which he recites a similar case to mine, but which lacked confirmation of other observers, and, as you all remember, was not given much credence, either by editors or by the profession generally; chiefly because it was thought an impossibility. The probabilities are that it was a genuine case of tubo-intersticial pregnancy.

In "Thomas on Diseases of Women," p. 767, we read: "Speigelberg reports a case of an ovum advancing to maturity in the tube. Intersticial pregnancy is much less frequent and less dangerous than the variety just mentioned. It is much more likely to advance to full term, and while it may produce death by rupture and discharge into the peritoneum, it may, as in my fourteenth case, discharge into the uterus and be expelled through the natural passages.

"Dr. Lenox Hodge once succeeded in recognizing the existence of such a case at full term, cut through the layer of perenchyma which shut the fœtus off from the uterus, and conducted the case to a successful issue."

Dr. A. D. Rockwell, of New York, says in "American System of Obstetrics and Gynecology," Vol. 1, pp. 405 and 406: "That the destruction of fœtal life could be easily effected by electricity admitted of no doubt, but whether it was possible to do this, without, in any way injuring the mother, was a question that could be determined only by experimental effort. This opportunity was offered, some years ago, by a case in the practice of Dr. Charles McBurney, when the method was suggested by Dr. T. G. Thomas, and I was asked to superintend the operation.

"The case was one of tubo-intersticial pregnancy at the third month, and terminated favorably by the expulsion of the fœtus and placenta through the uterus. Subsequently, eleven other cases fell under my observation, all of which I submitted to similar treatment, and with results entirely satisfactory."

Thus it will be seen, from the above references to these eminent authorities that Dr. Lowry's case and my own were possible.

It is also a matter of legitimate inference that, as I used the battery in precisely the same manner as did Dr. Rockwell, without, however, the same purpose, that treatment may have occasioned the favorable termination of my case.

[Dr. Lowry's case was claimed to have reached full term, and the fœtus was not in the tube.—ED.]

